# Adenovirus-associated acute conjunctivitis in Beijing, China, 2011–2013

**DOI:** 10.1186/s12879-018-3014-z

**Published:** 2018-03-20

**Authors:** Jie Li, Xiaoyan Lu, Baoming Jiang, Yiwei Du, Yang Yang, Haikun Qian, Baiwei Liu, Changying Lin, Lei Jia, Lijuan Chen, Quanyi Wang

**Affiliations:** 1 Beijing Center for Disease Prevention and Control, No.16, Hepingli Middle Road, Beijing, 100013 People’s Republic of China; 2 Research Centre for Preventive Medicine of Beijing, No.16, Hepingli Middle Road, Beijing, 100013 People’s Republic of China; 3 Centers for Disease Control and Prevention, 1600 Clifton Road NE, Atlanta, GA 30329 USA

**Keywords:** Acute conjunctivitis, Human adenovirus, HAdV type

## Abstract

**Background:**

Human adenovirus (HAdV)-associated acute conjunctivitis is a common infectious disease and causes significant morbidity among residents in Beijing, China. However, little is known about the epidemiology and type distribution of acute adenoviral conjunctivitis in Beijing.

**Methods:**

Acute conjunctivitis surveillance was conducted in 18 hospitals in Beijing from July through October during 2011–2013. HAdVs were detected by PCR from eye swab and types were determined by partial hexon and fiber gene sequencing. Risk factors associated with adenoviral conjunctivitis were analyzed.

**Results:**

Of 876 conjunctivitis cases, 349 (39.8%) were HAdV positive. HAdV detection was most common in conjunctivitis patients aged 18–40 years; patients with contact history with a conjunctivitis case; patients with specimen collected on days 4–6 post symptom onset and patients who worked in food service as catering attendants. Fifteen types were identified among adenoviral conjunctivitis cases. Five HAdV types (HAdV-4, − 37, − 53, − 64 and − 8) accounted for 81.1% of all adenoviral conjunctivitis cases. HAdV-37, − 4 and − 53 were the most common types associated with adenoviral conjunctivitis in 2011, 2012 and 2013, respectively.

**Conclusion:**

Multiple HAdV types were associated with acute conjunctivitis in Beijing. Predominant types associated with adenoviral conjunctivitis circulating in Beijing varied from year to year.

## Background

Acute conjunctivitis is a very common medical condition [1]. Approximately 20–70% of infectious conjunctivitis is due to viruses and 65–90% of these are caused by human adenoviruses (HAdVs) [2–4]. HAdV conjunctival infections can result in diminished vision and prolonged discomfort and occasional severe sequel [4]. HAdV-associated conjunctivitis has caused significant morbidity and medical costs [4–6].

HAdVs belong to the *Adenoviridae* family, *Mastadenovirus* genus, and are further divided into 7 species (A-G) [7]. The first 51 recognized serotypes were described based on their immunologic, biologic and biochemical characteristics and subsequent types (52–68) were classified based on genomics criteria [8–14]. HAdV-3 and -7 of species B, HAdV-8, − 19, and − 37 of species D, HAdV-4 of species E and recently identified genotypes HAdV-53, − 54, − 56 and − 64 of species D are also associated with eye infections [9, 15–21].

A previous study found that HAdV was the most prevalent pathogen associated with acute conjunctivitis in Beijing [1]. However, little is known about the epidemiology and type distribution of adenoviral conjunctivitis in Beijing. The aim of this study was to identify HAdV types associated with the acute adenoviral conjunctivitis in Beijing and describe the demographic, epidemiologic and risk factors of HAdV-associated conjunctivitis.

## Methods

### Definitions

Acute conjunctivitis, also referred as pink eye, was defined as any inflammation of the conjunctiva, which is generally characterized by a reddening of the eye with symptoms that may include pain, itching, and foreign body sensation accompanied by tearing or discharge [4]. Contact history was defined that a case lived, worked or studied with a conjunctivitis case or had close contact with a conjunctivitis case during the 14 days before the onset of acute conjunctivitis [7].

### Sample and data collection

From July to October during 2011–2013, a total of 18 hospitals out of 113 tertiary hospitals and 155 secondary hospitals from 18 districts in Beijing were selected to participate in the acute conjunctivitis surveillance program which was designed and managed by Beijing Center for Disease Control and Prevention (CDC). The 18 hospitals are either the largest or have the most patient visits in the area. Each month, the first 5 outpatients with acute conjunctivitis diagnosed by ophthalmologists from each surveillance hospital were invited to participate in this study. The median number of clinically diagnosed acute conjunctivitis cases at enrolled hospitals in July, August, September and October was 25.0 (IQR: 18.3–65.0), 27.5 (IQR: 15.8–61.8), 25.5 (IQR: 9–49.5) and 19.5 (IQR: 10–39.8), respectively. Questionnaires were designed and used by trained personnel at each site to collect demographic information, clinical information and epidemiological information. Informed consent was obtained from all participants or their guardians according to the study protocol. Eye swab samples were collected in minimum essential media (MEM) and stored at 4 °C until transfer to Beijing CDC for laboratory test.

### Sample processing

Total nucleic acid was extracted from 200 μl of sample using a MagNA Pure LC 2.0 nucleic extraction system (Roche Diagnostics Ltd. Rotkreuz Switzerland) with the MagNA Pure LC Total Nucleic Acid Isolation Kit-Large Volume following the manufacturer’s instructions.

### HAdV detection and identification

HAdV detection was performed by PCR assay using HotStar Taq Master Mix Kit (Qiagen GmbH, Hilden, Germany) with previously published primers [22]. HAdV positive specimens were also tested for herpes simplex virus (HSV), *Chlamydiatrachomatis* (Chlamydia), and the enteroviruses, Coxsackie A24 variant (CoxA24v) and enterovirus type 70 (EVA70) using previously described methods [22, 23]. For typing, HAdV hexon gene hypervariable regions 1–6 (HVRs1–6) were amplified using a nested PCR method with Premix Ex Taq™ DNA Polymerase Hot Start Version (Takara, Japan) with primers and cycling conditions [24]. Amplicon sequencing was performed in both directions using the amplification primers with the ABI Prism® Bigdye™ Terminator Cycle Sequencing Ready Reaction Kit Ver. 3.1 on an ABI 3100 DNA Sequencer (ThermoFicher). Sequencher™ 3.1.1 software (Gene Codes, AnnArbor, MI) was used for sequence assembly and editing. For some HAdV positive specimens that could not be accurately typed by partial hexon gene sequencing, the fiber gene was sequenced using previously described primers and cycling conditions [25]. Pre-PCR, template addition and post-PCR procedures were performed in separate rooms to prevent cross contamination. Positive and negative controls were used in all PCR runs.

Predicted amino acid sequences of partial hexon gene sequences obtained in this study together with 68 prototype strains available in GenBank were aligned using ClustalW implemented in BioEdit 7.1.3. Phylogenetic trees were constructed for aligned amino acid sequences using the neighbor joining method implemented in MEGA6 software. Type determinations were based on the highest percentage identity scores and bootstrap support values (1000 bootstrap replicates) obtained by phylogenetic analysis of hexon and fiber sequence alignments comparing “unknown” and prototype strain sequences.

### Statistical methods

Statistical analysis was performed using SPSS 17 software (IBM SPSS, Inc., Chicago, IL, USA). Comparisons of proportions and statistical significance were performed using the Chi-square test. A multiple logistic regression analysis was used to examine the multivariate-adjusted odds ratios (OR) for risk factors that were significant in univariate analysis. The enter method was used for screening of variables, and goodness-of-fit tests (Hosmer-Lemeshow) were performed on the logistic model. A *p*-value< 0.05 (2-sided significance test) was considered statistically significant in the above analyses.

### Ethics statement

This study was conducted in compliance with the Declaration of Helsinki, and the protocol was approved by the Human Research Ethics Committee of Beijing CDC.

## Results

### Demographic characteristics of conjunctivitis cases

Of 876 acute conjunctivitis cases enrolled in this study, 290 (33.1%), 283 (32.3%) and 303 (34.6) were obtained from 2011, 2012 and 2013, respectively. Of the study participants, 484 (55.3%) were male. Ages ranged from 5 months to 86 years with a median age of 32 years. HAdV was detected in 119 (41.0%), 125 (44.2%) and 105 (34.7%) of swab samples collected during each of the 3 years, respectively.

### Type distribution of HAdV-associated acute conjunctivitis

Among 349 eye swabs positive for HAdV, 342 (98.0%) were successfully typed. Seven (2.0%) samples were not successfully typed due to low HAdV viral load. HAdV types varied during the surveillance period of the three study years (Table 1). In 2011, the major types identified from conjunctival swab samples were HAdV-37 (25/119, 21.0%) and HAdV-64 (23/119, 19.3%). In 2012, HAdV-4 (34/125, 27.2%) became predominant followed by HAdV-37 (24/125, 19.2%). In 2013, HAdV-53 predominated (22/105, 21.0%) followed by HAdV-8 (18/105, 17.1%). In the study period, HAdV-4 (65/349, 18.6%), HAdV-37 (61/349, 17.5%) and HAdV-53 (59/349, 16.9%) were the three most common types associated with acute conjunctivitis.

**Table 1:** Identification of HAdV types in outpatients with acute conjunctivitis in Beijing, 2011–2013

		2011 (*N* = 119)	2012 (*N* = 125)	2013 (*N* = 105)	Total (*N* = 349)
Species	Type	n(%)	n(%)	n(%)	n(%)
B	HAdV-3	10(8.4)	10((8.0)	6(5.7)	26(7.4)
	HAdV-7	10((8.4)	7(5.6)	2(1.9)	19(5.4)
	HAdV-11	1(0.8)	1(0.8)	2(1.9)	4(1.1)
	HAdV-14	0(0.0)	0(0.0)	1(1.0)	1(0.3)
C	HAdV-1	0(0.0)	2(1.6)	0(0.0)	2(0.6)
	HAdV-2	0(0.0)	0(0.0)	2(1.9)	2(0.6)
	HAdV-5	1(0.8)	0(0.0)	0(0.0)	1(0.3)
D	HAdV-37	25(21.0)	24(19.2)	12(11.4)	61(17.5)
	HAdV-64	23(19.3)	13(10.4)	15(14.3)	51(14.6)
	HAdV-8	13(10.9)	16(12.8)	18(17.1)	47(13.5)
	HAdV-42	0(0.0)	0(0.0)	1(1.0)	1(0.3)
	HAdV-48	0(0.0)	0(0.0)	2(1.9)	2(0.6)
	HAdV-53	21(17.6)	16(12.8)	22(21.0)	59(16.9)
	HAdV-56	0(0.0)	1(0.8)	0(0.0)	1(0.3)
E	HAdV-4	14(11.8)	34(27.2)	17(16.2)	65(18.6)
	Untyped	1(0.8)	1(0.8)	5(4.8)	7(2.0)

### Age and gender characteristics of patients with and without HAdV infection

Among the 349 HAdV positive conjunctivitis patients, 203 were male and 146 were female with a male/female ratio of 1.39:1. No significant difference was observed in gender between HAdV positive cases and negative cases (*p* = 0.158) (Table 2).

**Table 2:** Factors associated with HAdV infection in patients with acute conjunctivitis

Category	Conjunctivitis cases, N	HAdV Positive n (%)	*p -* value^e^
Gender			
Male	484	203 (41.9)	0.158
Female	392	146 (37.2)	
Age group (year)^a^			
0–6	36	14 (38.9)	**< 0.001**
7–17	58	23 (39.7)	
18–40	479	222 (46.3)	
41–65	233	78 (33.5)	
≥ 66	70	12 (17.1)	
Occupation			
Children in daycare	15	6 (40.0)	**< 0.001**
Children stay-at-home	17	8 (47.1)	
Student	89	32 (36.0)	
Teacher	21	9 (42.9)	
Food and beverage server	26	16 (61.5)	
Commercial service personnel	83	36 (43.4)	
Physician	20	3 (15.0)	
Laborer	136	71(52.2)	
Farmer	85	24 (28.2)	
Government employee	87	34 (39.1)	
Retired people	100	22 (22.0)	
Unemployed	84	33 (39.3)	
Other	113	55 (48.7)	
Geographic distribution			
Urban	404	160 (39.6)	0.859
Suburban	472	189 (40.0)	
Student^b^			
Summer vacation (Jul-Aug)	53	19 (35.8)	0.980
School term (Sep-Oct)	36	13 (36.1)	
Contact history with a conjunctivitis case^c^			
Yes	83	51 (61.4)	**< 0.001**
No	659	240 (36.4)	
Sampling day after symptom onset^d^			
1st	156	46 (29.5)	**< 0.001**
2nd	191	51 (26.7)	
3rd	155	60 (38.7)	
4th	131	68 (51.9)	
5th	70	42 (60.0)	
6th	39	22 (56.4)	
7th	25	11 (44.0)	
≥ 8th	88	28 (31.8)	

^a^Age was divided into 5 groups according to age segmentation in China

^b^A total of 89 students were included in this analysis

^c^134 patients were not sure if they had a contact history and were excluded from analysis. A total of 742 patients were included in this analysis

^d^21 mixed-infection cases were observed in this study and was excluded from analysis. A total of 855 patients were included in the analysis.Associations between risk factors and HAdV infection were done by univariate analysis

^e^*P*-value was conducted by Pearson’s χ2. The bold values means significant difference was obtained between or among compared groups

The median age of HAdV positive and negative acute conjunctivitis cases were 34 years (Range: 0–78) and 30 years (Range: 0–86), respectively. HAdV detection rate among conjunctivitis cases increased from 7 to 17 years of age (39.7%), reached the peak in the age group of 18–40 years old (46.3%), and then declined with age dramatically. Acute conjunctivitis patients aged ≥66 years had the lowest HAdV detection rate (17.1%). Multiple logistic regression analysis showed that patients aged 18–40 years were more likely to have adenoviral conjunctivitis compared with patients aged ≥66 years (Table 3).

**Table 3:** Multiple logistic regression analysis of factors associated with HAdV infection in patients with acute conjunctivitis

Variables	*p-value*	aOR^b^ (95% CI)
Gender	0.816	1.037 (0.762–1.413)
Age group (year)		
0–6	0.832	0.769 (0.068–8.695)
7–17	0.079	2.692 (0.891–8.137)
18–40	**0.020**	**2.599 (1.166–5.794)**
41–65	0.129	1.802 (0.842–3.857)
≥ 66	Ref	
Occupations		
Preschool children and kindergarten children	0.060	13.533 (0.895–204.588)
Student	0.127	3.010 (0.732–12.382)
Teacher	0.075	4.147 (0.867–19.842)
Food and beverage server	**0.005**	**8.713 (1.918–39.576)**
Commercial service personnel	**0.031**	**4.438 (1.148–17.155)**
Laborer	**0.010**	**5.719 (1.52–21.518)**
Farmer	0.138	2.845 (0.715–11.327)
Government employee	0.074	3.433 (0.889–13.257)
Retired people	0.235	2.369 (0.571–9.839)
Unemployed	0.064	3.585 (0.927–13.874)
Others	**0.023**	**4.692 (1.238–17.786)**
Physician	Ref	
Sampling day after symptom onset		
1st and 2nd	Ref	
3rd	**0.042**	**1.547 (1.016–2.355)**
4th	**< 0.001**	**2.819 (1.810–4.391)**
5th	**< 0.001**	**3.817 (2.171–6.713)**
6th	**0.001**	**3.265 (1.583–6.736)**
7th	0.128	1.956 (0.825–4.640)
≥ 8th	0.736	1.093 (0.644–1.865)
Contact history with a conjunctivitis case		
Not sure whether there was a contact history	0.092	1.423 (0.944–2.145)
Yes	**0.001**	2.380 (1.420–3.990)
No	Ref	

^a^patients with mixed-infection were excluded

^b^multivariate-adjusted OR (aOR) calculated after controlling for other significant factors and gender. The bold values means significant difference was obtained when this group was compared with the reference group

### Occupation and geographic distribution

HAdV detection rate among acute conjunctivitis patients was significantly different among 13 occupations (*P* < 0.001) (Table 2). Acute conjunctivitis caused by HAdV infection was lowest among physicians (15.0%) and highest among food and beverage servers (61.54%). Multiple logistic regression analysis showed that food and beverage servers (*p* = 0.005), followed by laborers (*p* = 0.01) and commercial service personnel (*p* = 0.031) were more likely to have adenoviral conjunctivitis compared with physician (Table 3). No significant difference of HAdV-associated conjunctivitis was found between patients living in urban or suburban settings (*P* = 0.859) or between students at school or on vacation (*p* = 0.980) (Table 2).

### Contact history and sampling day

Among the 83 acute conjunctivitis patients that had contact history with a conjunctivitis case, 51 (61.4%) had HAdV detected from eye swabs. By contrast, 36.4% (240/659) of the cases without contact history had HAdV detection. The difference in HAdV detection rate among acute conjunctivitis patients with and without contact history was significant (*p* < 0.001) (Table 2). After controlling for other significant factors, persons with contact history with a conjunctivitis case were more likely to have adenoviral conjunctivitis than those without contact history (Table 3).

Of the 349 HAdV positive eye swabs, a mixed infection was observed in 21 specimens. The most common pathogen co-detected with HAdV was Chlamydia (*n* = 12) followed by HSV (*n* = 6) and CoxA24 (*n* = 4). After removing the influence from mixed infections, we found that the HAdV detection rate in eye swabs was 29.5% on the first day of disease onset, then increased to 38.7% at day 3, and peaked at day 5 (60.0%) and 6 (56.4%). A significant difference was shown in the HAdV detection rate among different sample collection days (*p* < 0.001) (Table 2). Multiple logistic regression analysis showed that HAdV detection rate among acute adenoviral conjunctivitis patients was significant higher from the 3th to 6th day of sampling compared with the first two days (Table 3).

### HAdV type distribution between genders and among age groups

Analysis of the prevalence and demographic distribution of the five major HAdV types (HAdV-4, − 37, − 53, − 64 and − 8) found significant difference in HAdV type distribution between two genders (*p* = 0.013) (Table 4). HAdV-4 (37/114, 29.8%) and HAdV-37 (44/169, 26.0%) were the most common type in female and male patients, respectively. HAdV-4 positive rate among female was significantly higher than male (*p* = 0.003). No significant difference of HAdV-4 positive rate was found between children and older age groups (*p* = 0.320). No significant difference in five major HAdV types distribution was observed among different age groups (*P* > 0.05).

**Table 4:** Analysis of the demographic information among different HAdV types

Characteristics (*N* = 283)	HAdV-4 (*N* = 64) n (%)	HAdV-37 (*N* = 62) n (%)	HAdV-53 (*N* = 59) n (%)	HAdV-64 (*N* = 51) n (%)	HAdV-8 (*N* = 47) n (%)	*p-*value^a^
Gender						
Male (*n* = 169)	27 (16.0)	44 (26.0)	39 (23.1)	32 (18.9)	27 (16.0)	**0.013**
Female (*n* = 114)	37 (32.5)	18 (15.8)	20 (17.5)	19 (16.7)	20 (17.5)	
Age group(year)						
0–6 (*n* = 11)	5 (45.5)	0 (0.0)	2 (18.2)	2 (18.2)	2(18.2)	0.228
7–17 (*n* = 18)	8 (44.4)	3 (16.7)	2 (11.1)	1 (5.6)	4 (22.2)	0.157
18–40 (*n* = 181)	38 (21.0)	43 (23.8)	37 (20.4)	34(18.8)	29 (16.0)	0.796
41–65 (*n* = 68)	11 (16.2)	14 (20.6)	17 (25.0)	14 (20.6)	12 (17.6)	0.590
≥ 66 (*n* = 5)	2 (40.0)	2 (40.0)	1 (20.0)	0(0.0)	0 (0.0)	0.951

^a^*P-*value was conducted by Pearson’s χ^2^, Fisher’s exact test. The bold values means statistically significance was obtained among compared groups

## Discussion

This study was conducted from July through October over 3 consecutive years and 876 outpatients were enrolled in the study to determine the etiology of acute conjunctivitis in Beijing. Our results showed that HAdV was responsible for 39.8% of acute conjunctivitis cases. This result was lower than that reported in Japan during 2005–2006 (82%) [3], Pakistan (75%) [2] and Brazil during 2004 to 2007 (60%) [26].

Adenoviral conjunctivitis was mainly caused by HAdV types 3, 4, 7, 8, 11, 37 and 64 which have high affinity to the conjunctival epithelium [15–19, 27–29]. In our study, a total of 15 HAdV types were identified. HAdV-4, HAdV-37 and HAdV-53 were the most common types followed by HAdV-64 and HAdV-8. This is different from previously reported studies from other countries. From 2005 to 2006 in Tokyo [3], 2000–2013 in Tunisia [30] and 2006–2010 in Turkey [31], the most common type causing adenoviral conjunctivitis was HAdV-8. Although both HAdV-4 and HAdV-8 were associated with acute conjunctivitis, HAdV-4 caused broad symptoms ranging from pharyngoconjunctival fever to adenoviral keratoconjunctivitis [32], whereas HAdV-8 was frequently associated with subepithelial corneal opacities without systemic syndrome [33]. The prevalence of membranous conjuntivitis was high (83%) among patients infected with HAd-37 which ranked the second in Beijing [33]. HAdV-53, − 56 and − 64 are novel types which were characterized using genomics and bioinformatics [14, 34]. In this study approximately 6.5% acute conjunctivitis was associated with HAdV-53. Similar to our findings, HAdV-53 associated epidemic keraconjunctivitis (EKC) has been identified in Japan and Germany and has recently ranked the third in Japan following HAdV-37, HAdV-54 [19]. Only one case in this study was identified as HAdV-56 which also caused keratoconjunctivitis [34], urethritis [21, 35] and caused an EKC outbreak in China in 2012 [20]. HAdV-64 originating from a recombination between HAdV-19p, HAdV-37, and HAdV-22 has been closely associated with keratoconjunctivitis [17]. In this study HAdV-64 was the major type associated with acute conjunctivitis in Beijing which was accordant with a previous study [36].

HAdVs are resistant to enviromental influences and can remain infectious on surfaces for up to 4–5 weeks [4]. HAdVs can be easily transmitted through fomites contaminated with infectious body fluids. In this study, patients who had contact history with a conjunctivitis case were 2.38 times of being infected with HAdV than acute conjunctivitis patients without contact history. These results suggest that exposure to infected person can greatly increase the risk of infection. Thus, it is imperative to recommend good hand washing, avoiding sharing personal items and advocating isolation of the infected patients to prevent the spread of this disease [37].

We found that patients with HAdV infections in age group of 18–40 years old had 2.6 times the chance of being infected than those ≥66 years old. Our observations were similar to an EKC outbreak in Germany in which all the infected patients were between 20 and 29 years old [38]. In our study, the oldest age group has the lowest HAdV detection rate probabaly because the elderly stay at home more and are less likely to contact HAdV infected patients or contaminated enviroment. Since people of 18–40 years old are at high risk to be infected with acute conjunctivistis, health education on how to avoid catching this disease should be encouraged among them.

We also observed significant difference in HAdV detection among patients with conjunctivitis in 13 occupations. Among patients with acute adenoviral conjunctivitis, food and beverage workers were more likely than physicians to have HAdV as the etiology. Food and beverage workers generally have low educational level in China. According to a previous study [39], people with lower education level demonstrate lower health literacy. Since food and beverage workers are exposed to many people every day, with poor health literacy, they are more likely to be infected with HAdV. Once they get infected, they are likely to infect their colleagues and customers. To prevent and control of adenoviral conjunctivitis, food and beverage workers should be the focus of intervention. It is important for them to keep good personal hygiene habit to prevent them from being infected. Moreover, good professional responsibility should be hold. Once they get infected, they should stop working until they are fully recovered. According to the result in this study, the positive rate were the highest at the 4th, 5th and 6th day after symptom onset, we suggest that patients should be isolated in the high concentration virus excretion stage to avoid transmitting virus to other people.

A previous study reported that viral incubation time was 2–14 days and patients might remain infectious for 10–14 days after the onset of symptoms [7]. In our study, the HAdV detection rate differed significantly with the sample collection day after controlling for other significant factors. Samples collected on the3^th^, 4th, 5th and 6th day after symptom onset might be more likely to be positive than those collected on the first two days from onset. This result is consistent with the observations that secretory IgA and IgG against HAdV can be detected in serum and nasal secretions at approximately 7 days post-infection [40, 41]. Since the virus concentration in the eye would start low in the first two days, peak on the 5th or 6th day and then decline grandually with increased antibody titer, we suggest that negtive result on an early sample should be followed by a second sample to increase the likelihood of HAdV detection. On the other hand, infected patients should be isolated especially in the high concentration virus excretion stage in order to limit infection source and prevent outbreaks in large urban areas like Beijing.

This study has several limitations. First, acute conjunctivitis surveillance was only conducted from July to October in three years, thus we were not able to perform year-round assessment of HAdV in association with acute conjunctivitis in Beijing. Second, unavailability of detailed clinical information made it impossible to establish association between HAdV types and different clinical presentations of viral conjunctivites. Third, since HAdV was detected only by PCR from eye swab and viral culture was not perfomed, we were not sure whether the virus detected on the 4th- 6th day after symptom onset remained infectious, we suggest that patients with HAdV infection during this peak shedding period were most contagious.

## Conclusions

In conclusion, multiple HAdV types were associated with acute conjunctivitis among residents in Beijing. Infected person should be isolated during the period of virus excretion, especially 3–6 days after symptom onset. Good hygenic habits and disinfection of potentially contaminated environmental surfaces should be routinely practiced to reduce the spread of HAdV-associated conjunctivitis.

## Abbreviations

CDC: Center for Disease Control and Prevention
Chlamydia: *Chlamydiatrachomatis*
CoxA24v: Coxsackie A24 variant
EKC: epidemic keraconjunctivitis
HAdV: Human adenovirus
HSV: herpes simplex virus
IQR: interquartile range
MEM: minimum essential media
OR: odds ratios
